# Complexity of the Genetic Background of Oncogenesis in Ovarian Cancer—Genetic Instability and Clinical Implications

**DOI:** 10.3390/cells13040345

**Published:** 2024-02-15

**Authors:** Marek Murawski, Adam Jagodziński, Aleksandra Bielawska-Pohl, Aleksandra Klimczak

**Affiliations:** 1 1st Clinical Department of Gynecology and Obstetrics, Wroclaw Medical University, 50-367 Wroclaw, Poland; marek.murawski@umw.edu.pl; 2 Laboratory of Biology of Stem and Neoplastic Cells, Hirszfeld Institute of Immunology and Experimental Therapy, Polish Academy of Sciences, 53-114 Wroclaw, Poland; aleksandra.bielawska-pohl@hirszfeld.pl (A.B.-P.); aleksandra.klimczak@hirszfeld.pl (A.K.)

**Keywords:** genetic instability, oncogenesis, ovarian cancer, RRSO, *TP53* mutations, *BRCA1* mutations, *BRCA2* mutations, *BRAF* mutations, *RAS* mutations, *ARID1A* mutations, *PIK3CA* mutations, *PTEN* mutations, *CTNNB1* mutations, c-myc amplification

## Abstract

Ovarian cancer is a leading cause of death among women with gynecological cancers, and is often diagnosed at advanced stages, leading to poor outcomes. This review explores genetic aspects of high-grade serous, endometrioid, and clear-cell ovarian carcinomas, emphasizing personalized treatment approaches. Specific mutations such as *TP53* in high-grade serous and *BRAF/KRAS* in low-grade serous carcinomas highlight the need for tailored therapies. Varying mutation prevalence across subtypes, including *BRCA1/2*, *PTEN*, *PIK3CA*, *CTNNB1*, and c-myc amplification, offers potential therapeutic targets. This review underscores *TP53*’s pivotal role and advocates p53 immunohistochemical staining for mutational analysis. *BRCA1/2* mutations’ significance as genetic risk factors and their relevance in PARP inhibitor therapy are discussed, emphasizing the importance of genetic testing. This review also addresses the paradoxical better prognosis linked to *KRAS* and *BRAF* mutations in ovarian cancer. *ARID1A*, *PIK3CA*, and *PTEN* alterations in platinum resistance contribute to the genetic landscape. Therapeutic strategies, like restoring WT p53 function and exploring PI3K/AKT/mTOR inhibitors, are considered. The evolving understanding of genetic factors in ovarian carcinomas supports tailored therapeutic approaches based on individual tumor genetic profiles. Ongoing research shows promise for advancing personalized treatments and refining genetic testing in neoplastic diseases, including ovarian cancer. Clinical genetic screening tests can identify women at increased risk, guiding predictive cancer risk-reducing surgery.

## 1. Introduction

Ovarian cancer (OC) is the malignant neoplasm of the female reproductive organs associated with the highest mortality rate [1,2] and ranks eighth in terms of the incidence of malignant tumors worldwide according to 2020 data [2]. The population risk of developing OC is 1.3%. Ovarian cancer includes a heterogeneous group of malignant tumors, within which there are differences regarding, among other things, etiology and molecular biology. Most ovarian malignant tumors are of epithelial origin, with serous ovarian cancer being the most common. The incidence of OC is estimated at 2.5% among malignant tumors in women; however, it accounts for 5% of deaths in that group of patients. Ovarian cancer is most commonly diagnosed in clinical stages III and IV. The World Health Organization divides ovarian cancers into several groups, within which benign, borderline, and malignant cancers can be distinguished. From a pathological point of view, a distinction is made between epithelial (serous, mucinous, endometrioid, clear-cell, Brenner-type, serous-mucinous, undifferentiated) and mesenchymal, germ cell and sex-cord stromal tumors. Clinically, epithelial ovarian cancers can be divided into type I and type II. The first group represents 25% of cases and is characterized by slow growth, while the second group, which corresponds to 75% of patients, is associated with greater aggressiveness and a higher degree of clinical advancement at diagnosis. Histopathologically, low-grade serous carcinomas (LGSC) and high-grade serous carcinomas (HGSC) can be distinguished among epithelial ovarian cancers. Both groups are characterized by the presence of different genetic abnormalities and the understanding and explanation of the natural processes leading to carcinogenesis, and the course of the disease and treatment options are of key importance for improving diagnostic and therapeutic possibilities in OC.

As our understanding deepens regarding the genes associated with the homologous recombination pathway and the clinical implications of their mutations, there is potential for customizing cancer treatment and refining the utilization of targeted therapies. Additionally, these advancements can enhance the identification of high-risk families, subsequently boosting screening and prevention strategies. Therefore, advancement in this direction should be approached with a sense of urgency.

Differences in tumor genetic profiles among patients with high-grade serous, endometrioid, and clear-cell ovarian carcinomas have been widely confirmed. These discrepancies hold important implications for customizing therapies based on individual patient needs and for identifying suitable participants for targeted clinical studies. This review underscores the potential of comprehensive genetic testing while providing valuable insights for selecting specific genes to include in testing panels. Together, the decade-long findings contribute to advancing personalized treatment strategies and optimizing genetic testing approaches within the realm of ovarian carcinomas.

One thing is certain: while there are similarities in the patients who present with advanced-stage disease, their histologic and molecular features are entirely different. High-grade serous carcinoma is associated with *TP53* mutations, whereas low-grade serous carcinomas are associated with *BRAF* and *KRAS* mutations. Endometrioid and clear-cell carcinomas are typically recognized at early stage of the disease and are frequently associated with endometriosis. Mucinous carcinomas typically present as large unilateral masses and often show areas of mucinous cystadenoma and mucinous borderline tumor. It must be emphasized that primary mucinous carcinomas are uncommon tumors, and metastasis from other sites such as the appendix, colon, and stomach therefore must always be considered in the differential diagnosis.

## 2. Genetic Landscape of Epithelial Ovarian Cancer: Implications for Prognosis and Targeted Therapies

Numerous investigations have consistently highlighted a considerable correlation between gene mutations and the clinical phenotype of various cancers. This suggests the potential utility of gene mutation loci as both prognostic indicators and consequently targets for therapeutic interventions/approaches [3]. Epithelial ovarian cancer exhibits a strong association with a few frequently reported gene mutations, namely *TP53*, *BRCA1/2*, *BRAF*, *RAS*, *ARID1A*, *PIK3CA*, *PTEN*, and *CTNNB1*. They could be divided into two main subgroups of alternations: DNA repair-related genes (*BRCA1/2*, *PTEN*) and genes involved in cell signaling pathways as well as the cell cycle (*PI3K/AKT/mTOR*, *RAS/MAPK*). As mentioned above, the prevalence of these mutations varies across distinct subtypes of epithelial ovarian cancer [4,5,6]. *TP53* mutations are predominant in high-grade serous ovarian cancer (HGSOC), with an increased mutation rate reaching 55%. Hereditary ovarian cancer is primarily linked to *BRCA1/2* genes, and the mutation rate escalates to 40% in recurrent HGSOC cases. *PIK3CA* mutations exhibit heightened prevalence in ovarian clear cell carcinoma (OCCC) and endometrioid ovarian cancer (EnOC) associated with endometriosis. Meanwhile, *BRAF* mutations play a pivotal role in low-grade serous ovarian cancer (LGSOC). *PTEN* mutations are characteristic for endometrioid ovarian cancer (EnOC) and ovarian clear cell carcinoma (OCCC), while *CTNNB1* is characteristic for endometrioid ovarian cancer (EnOC). The potential mechanisms linking mutations to ovarian cancer (OC) encompass several aspects. These include the loss of function of genes that regulate tumor suppression, abnormalities in DNA repair genes, disruptions in apoptosis (programmed cell death), gain of function in oncogenes, and epigenetic inactivation [4].

Nowadays, it is well known that both oncogenes (genes that promote cell growth and division) and tumor suppressor genes (TSGs, which regulate the cell cycle and prevent excessive cell growth) play crucial roles in the cancerous process. The knowledge that approximately 23% of ovarian carcinomas have a hereditary predisposition allows to use them as biomarkers in the clinical trials and associate them with better outcomes. Therefore, in this review, we summarize recent developments in the therapeutic approaches targeting ovarian cancer, specifically tailored to the mutations found in these genes.

### 2.1. TP53—Tumor Suppressor Gene That Encodes p53 Protein in Ovarian Cancer

*TP53*, often referred to as “the guardian of the genome” or the “cellular gatekeeper”, plays a crucial role in tumor suppression, being a checkpoint molecule. It achieves this by regulating the expression of downstream genes, triggering a range of cellular responses [6]. These responses include inducing cell cycle arrest or apoptosis in response to various types of stress, such as nutrient deprivation, telomere erosion, hypoxia, DNA damage, ribosomal stress, and oncogene activation [7,8,9,10]. The prevalence of *TP53* gene alterations appears to rise with increasing staging systems defined by FIGO [11]. The loss of the proper function of the p53 protein plays a key role in the development of a large group of cancers [7]. *TP53* mutations can manifest in various ways; some mutations may result in the loss or reduced activity of the wild-type (WT) p53 function (deletion or missense mutations), and others can give rise to new proteins with novel functions, referred to as gain-of-function (GOF) mutations. The majority of *TP53* mutations within ovarian cancer patients are single base-pair substitutions leading to the hyper-stabilization of the encoded protein [12]. Mutations are primarily localized to the highly conserved DNA binding domain, resulting in the inactivation of the WT p53 function [13]. In conclusion, with its acquired oncogenic capabilities, mutated p53 can be regarded as a renegade p53, and therefore it is essential to comprehend individual mutations in the gene before seeking associations between *TP53* mutations and patient outcomes. After a long debate, it was postulated that each *TP53* mutation has context-specific effects in response to different cancer therapeutics used [14,15].

The relationship between the presence of a *TP53* mutation and the abnormal nuclear accumulation of p53 protein is well-established in various cancers [8,16]. The use of p53 immunohistochemical (IHC) staining as a surrogate for *TP53* mutational analysis is a common and practical approach in pathology [8]. Nonsynonymous missense mutations in *TP53* can lead to the production of mutant p53 proteins with altered stability and function. One characteristic feature of many mutant p53 proteins is their prolonged half-life and resistance to degradation, resulting in their accumulation within the cell nucleus and specific distribution patterns within tissue samples. Therefore, in the context of ovarian cancer and other cancers, p53 IHC staining is frequently employed for several reasons: it is quick, easy to perform, and an inexpensive method.

Currently, the impact of restoring WT p53 function on inhibiting tumor growth can indeed be significant, but its effectiveness may vary depending on the stage of cancer progression. Taking all these facts into consideration, targeting mutated p53 protein may be a potential strategy for tumor-specific therapies precisely because these mutations are often absent or present at much lower levels in normal tissues. The concept is based on the idea of exploiting the specific genetic alterations present in cancer cells while sparing normal, healthy cells. Reports on the association between *TP53* status and disease progression or survival have emerged in the past decade. Nevertheless, conflicting conclusions have been drawn regarding the prognostic significance of *TP53* mutations in ovarian cancer [9]. All the discrepancies between reports may be explained by differences in the techniques used for the analysis of *TP53* status, patient sample size, biological and/or histological ovarian tumor subset analyses, different treatments of the patient population, different (modern) prognostic covariates used in the multivariate analyses, the inherent subjectivity in certain approaches, and publication bias. Generally, all these hypotheses are still based on insufficient analysis and inadequate methods and need to be revised.

Surprisingly, *TP53* mutations are less common in borderline tumors. The genetic alterations observed in borderline tumors may differ from those seen in invasive cancers [9]. Furthermore, as examined by Skilling et al. in their study on primary ovarian tumors, the prevalence of *TP53* gene mutations or overexpression is higher in serous primary ovarian cancers, with rates of 58% and 59%, respectively [17]. It is noteworthy that the frequency of these mutations varies among different subtypes of epithelial ovarian cancers, varying from 5% up to 96%, as introduced in review papers [4,18] and in The Cancer Genome Atlas (https://www.cancer.gov/ccg/research/genome-sequencing/tcga (accessed on 28 January 2024)).

Unfortunately, the *TP53* sequence alone cannot offer conclusive information to predict patients’ responses. Therefore, cellular effects of *TP53* mutations require exhaustive in vitro and in vivo studies to provide definitive information [13].

On the other hand, to understand the *TP53* mutations and their biological effects, the potential strategy involves the restoration of WT p53 function. This concept is well-founded, supported by both in vitro and in vivo research, along with evidence from various clinical trials [19,20,21,22]. These studies demonstrate that restoring WT p53 function leads to swift tumor regression in mice and prolonged survival in humans. Another approach is to inhibit the heat-shock protein HSP90, which chaperones many mutant p53 proteins [23,24]. Notably, there are still two significant challenges that hinder the realization of p53-based gene therapy which must be addressed: endogenous mutant p53 exerts a dominant negative inhibition of WT p53, and a high limitation of the delivery of p53-based gene therapy systems [25]. Therefore, re-engineered adenoviruses, next-generation p53-based therapies, or nanoparticle p53 therapies for the effective delivery of p53 remain an untapped field, with many other strategies still to be explored. The prognosis for numerous patients depends on the future of these therapies and the continued progress of ovarian cancer research.

### 2.2. BRCA1/2 (Breast Cancer 1/2)—Tumor Suppressor Genes

All the investigations clearly show that, not only for breast cancer, *BRCA1* and *BRCA2* mutations stand out as the foremost high-penetrance genetic risk factors for ovarian cancer. *BRCA1* and *BRCA2* are responsible for producing proteins that play vital roles in maintaining the integrity of our genetic material. These proteins are crucial for repairing damaged DNA, warranting the stability of the genome, and overseeing the orderly progression of cell division. Specifically, they excel in repairing double-stranded DNA breaks through a sophisticated process called homologous recombination (HR). This complex mechanism not only safeguards the genetic information within cells but also acts as a regulatory checkpoint to prevent errors during cell division. Indeed, the functions of *BRCA1* and *BRCA2* extend beyond DNA repair—they are guardians of genomic stability and custodians of the proper orchestration of cellular division processes [26]. The collected information highlights the significant role of *BRCA1* and *BRCA2* mutations in families with a history of two or more cases of ovarian cancer. Moreover, these mutations play a central role as the primary genetic risk factors for two well-defined syndromes: site-specific ovarian cancer and breast–ovarian cancer syndrome. The cumulative risks of developing ovarian cancer by the age of 70 differ among carriers of *BRCA1* and *BRCA2* mutations. For *BRCA1* carriers, the average cumulative risk was found to be 39% (ranging from 18% to 54%), whereas for *BRCA2* mutation carriers, the corresponding risk was 11% (with a range of 2.4% to 19%) [27]. A more recent meta-analysis and the last published data verified these findings, reporting ovarian cancer risks of 40% for *BRCA1* carriers and 18% for *BRCA2* carriers [28,29,30]. These statistics emphasize the distinct risk profiles associated with *BRCA1* and *BRCA2* mutations, providing valuable insights for risk assessment and clinical management. Unsurprisingly, the results of a meta-analysis encompassing 30 studies indicate elevated risks of developing cancer in various specific organs, including the stomach, pancreas, prostate, and colon [31]. Simultaneous literature reports indicate reductions in the risk of ovarian cancer in the general population after using oral contraceptives [32], parity [33], and breast-feeding [34].

Changes at the molecular level that disrupt the normal expression of *BRCA* genes can happen either through mutations in the gene itself or through a process called promoter methylation. Tumors become genetically highly unstable and undergo frequent and irregular changes, which can contribute to their development and progression [35]. It is noteworthy that there are some patients with phenotypic changes resulting from deficiencies in these genes, described as BRCAness [36]. These changes produce outcomes comparable to those observed in individuals with inherited *BRCA* mutations. Patients carrying these mutations may experience the development of ovarian cancer exhibiting characteristics and behaviors similar to tumors associated with *BRCA* mutations.

It is one of the few successes of clinical intervention for ovarian cancer patients that screening for *BRCA1/2* mutations is now routinely used in clinical practice. The defected *BRCA1/2* is the specific marker for the use of synthetic lethal-based PARPi (poly ADP ribose polymerase inhibitors) therapy in cancer treatment. This promising avenue of treatment is currently undergoing phase 1 and phase 2 clinical trials [37,38]. A meta-analysis published in 2021 demonstrated that incorporating PARP inhibitors (e.g., olaparib, niraparib) into the initial treatment regimen for advanced ovarian cancer patients with BRCA mutations significantly benefited progression-free survival compared to the time of relapse [39]. Based on the latest clinical trials, it is postulated that in advanced ovarian cancer, achieving a curative outcome is increasingly recognized as reasonable. Notably, in the first-line setting, the 7-year data from the SOLO-1 trail (olaparib) demonstrated a significant improvement in overall survival (OS) for patients with a BRCA mutation who received maintenance PARP inhibitor. Similarly, the 5-year results from the PAOLA-1 trail indicated a meaningful improvement in OS for patients with tumors testing positive for HR deficiency who received maintenance olaparib along with bevacizumab. However, the crucial challenge remains in identifying predictive factors that determine which patients will experience long-term remission with PARP inhibitor maintenance therapy [40]. The expectation is that therapeutic strategies based on PARP inhibition will prove effective in treating ovarian cancer, as well as breast cancer, characterized by the loss of *BRCA1/2* function, irrespective of whether the tumors are familial or sporadic in nature. Currently, several programs have been developed that enable accurate predictions of disease and the likelihood that a *BRCA1/2* mutation is present, based on a deleterious mutation in the family history. PARP inhibitors exploit synthetic lethality in *BRCA1/2* mutant ovarian cancer. The combination of HR deficiency (from BRCA mutations) and BER inhibition (by PARP inhibitors) leads to unrepaired double-stranded breaks, causing cell death. Moreover, *BRCA1/2*-mutant cancer cells are particularly sensitive to PARPi, creating a therapeutic window for selective cancer cell targeting [41]. However, it is important not to forget that *BRCA1* and *BRCA2* mutations are not very common, representing only a small fraction of the overall burden of ovarian cancer. Therefore, the American Society of Clinical Oncology Guidelines (2020) recommends germline testing of a multigene panel that should include *BRCA* and *non-BRCA*-related mutations in ovarian cancer patients. In addition to *BRCA1* and *BRCA2* mutations, it is crucial to recognize that various other genetic mutations, such as those in *RAD51C*, *ATM*, and *CHEK2*, can induce HR deficiency in ovarian cancer. These genetic alterations play a role in fostering genomic instability, heightening the likelihood of cancer onset. Understanding this broader spectrum of mutations is essential for a comprehensive assessment of ovarian cancer risk [42].

### 2.3. KRAS and BRAF Mutations

In various cancers, including colon cancer, melanoma, and thyroid cancer, tumors harboring mutations in the *KRAS* (Kirsten rat sarcoma viral oncogene homolog) and *BRAF* (encoding B-Raf protein) genes tend to be associated with a more aggressive behavior compared to wild-type tumors [43]. The *KRAS* gene codes for a protein involved in cell signaling pathways that regulate cell division and growth, and has been implicated as the most commonly mutated oncogene associated with human tumors [9]. The *BRAF* gene plays a crucial role in the *RAS*-*RAF*-*MEK*-*ERK* signaling pathway, which is involved in controlling cell proliferation. Moreover, the most common sites of mutation, V600E in *BRAF* and codon 12 in *KRAS*, have long been recognized to be oncogenic. Generally, *BRAF* and/or *KRAS* mutations can result in the constitutive activation of the mitogen-activated protein kinase (*MAPK*) pathway [44]. This continuous activation, in turn, initiates downstream protein kinases, nuclear proteins, and transcription factors, which may induce tumor development. The opposite situation was observed for women bearing ovarian cancer with mutations in *KRAS* and/or *BRAF* who had better prognosis than those without them [45]. This is the antithesis of what was detected with these oncogenic mutations in solid tumors. Both Grisham and Moujaber reported that for high-grade ovarian cancer, there is a loss rather than a gain of the *BRAF* and/or *KRAS* mutations, while low-grade ovarian cancers are predominantly characterized by these mutations [46,47]. It was proved that low-grade serous cancers that have the *BRAF* mutation have a better apparent clinical outcome [48]. Therefore, it is so important to understand the molecular basis and clinical behavior of ovarian cancer types to reduce the overtreatment of those women whose cancers are unlikely to recur [46,49]. Similar but different *BRAF* and *KRAS* mutation roles in oncogenesis generate potential implications for the treatment of invasive mucinous ovarian carcinoma and highlight a challenge in its response to conventional chemotherapy compared to high-grade serous carcinomas. The suggestion is that blocking *KRAS*/*BRAF* signaling in mucinous ovarian carcinoma could potentially offer a more effective therapeutic approach [50].

### 2.4. ARID1A—AT Rich Interactive Domain 1A (SWI-like; ARID1A)—And Its Crucial Role in Regulating Gene Expression That Drives Oncogenesis or Tumor Suppression

It acts as a “gatekeeper” by controlling the orderly progression of the cell cycle, ensuring that cell division occurs in a regulated manner. Simultaneously, it functions as a “caretaker” by safeguarding the stability of the genome, thereby preventing the occurrence of genetic instability. This dual role underscores the importance of *ARID1A* in maintaining the normal functioning of cells and protecting against the development of cancer by regulating cell cycles and preserving genomic integrity. Most *ARID1A* mutations observed are characterized as frame-shift or nonsense mutations, implying that *ARID1A* functions as a tumor suppressor. Certain mutations, such as *ARID1A* mutations, and alterations in the phosphatidylinositol 3-kinase (PI3K)/AKT/mTOR pathway, which are specific in ovarian clear cell carcinoma (OCCC) and endometrioid ovarian carcinoma (EnOC), may offer additional therapeutic targets in these clinical entities [51]. Researchers identified *ARID1A* mutations in 43–57% of ovarian clear cell carcinomas [52] and 30% of ovarian endometrioid carcinomas [53,54]. Few studies reported poorer progression-free survival in OCCC patients with *ARID1A* deficiency [55,56]. However, the existing literature consistently indicates that the overall survival in OCCC is not influenced by *ARID1A* status [57,58]. Additionally, endometrial carcinoma has been associated with deep myometrial invasion [59]. Moreover, it was shown that the mutation of *ARID1A* has a potential early-stage event in the oncogenic transformation of endometriosis cells, leading to the development of OCCC [60]. Knowing that multiple agents show synthetic lethality in an *ARID1A* mutant, there are several agents in clinical development trials [51]. Ongoing trials involving ovarian cancer patients provide the advanced clinical development of PI3K and PARP targeting compounds (http://clinicaltrials.gov (accessed on 28 January 2024)). Nowadays, a diverse range of compounds holds potential benefits in *ARID1A*-altered cancers, including immune checkpoint blockade (PDL-1) and inhibitors of mTOR, EZH2, histone deacetylases, ATR, and/or PARP. Moreover, it was postulated that *ARID1A* alterations may also mediate resistance to platinum chemotherapy and estrogen receptor degraders/modulators. Interestingly, *ARID1A* alterations seem to be associated with clinical resistance to platinum agents in ovarian cancer, as indicated by several studies [55,61,62]. These experiments clearly show that in ovarian cancer, *ARID1A* protein loss has led to the upregulation of multidrug resistance-associated protein 2 (MRP2) through transcriptional modifications. MRP2, in turn, facilitates the ATP-dependent active membrane export of platinum agents, suggesting a potential mechanism for platinum resistance [61,63].

### 2.5. PIK3CA (Phosphoinositide-3-Kinase) Mutation Phosphatidylinositol-4,5-Bisphosphate 3-Kinase, Catalytic Subunit Alpha

Phosphatidylinositol 3′-kinases are lipid kinases that play crucial roles in neoplasia, contributing significantly to the development and progression of cancer which initiates the malignant transformation of endometriosis [64,65]. Activated *PIK3CA*/AKT regulates the expression of several target genes and inhibits apoptosis by promoting cell proliferation. Its activation directly stabilizes the activity of the lipid phosphatase *PTEN* (phosphatase and tensin homolog), which serves as a negative regulator of the *PIK3CA*/AKT pathway [66]. *PIK3CA* mutation or gene amplification was detected in 30% of all ovarian cancers and 45% of the endometrioid and clear cell subtypes [67,68,69]. Mutations in *PIK3CA* lead to increased activity of phosphatidylinositol 3′-kinase, promoting enhanced cell survival, motility, and progression through the cell cycle, and were linked to a more favorable prognosis. In the ovarian-carcinoma-investigated patient group, the scientists argue against a simple linear model of *PIK3CA* gain/amplification followed by PI3K activation and consecutive AKT phosphorylation [70]. Moreover, PI3K activation did not serve as a predictive marker for the sensitivity of ovarian clear cell carcinoma investigated in Japanese patients cohort to *PIK3CA*/AKT/mTOR inhibitors [71]. Some evidence shows that only the combined use of *PIK3CA* inhibitor (BKM120) and PARP inhibitor (olaparib) may prove effective in treating ovarian cancers with a wider range of cancer-associated genetic alterations [72]. Delving into the topic, some data support the role of *PIK3CA* amplification as an independent factor in predicting the response to chemotherapy in ovarian cancer patients [73,74]. This discovery suggests that therapeutic intervention with specific kinase inhibitors might enhance chemotherapy sensitivity in ovarian carcinomas with *PIK3CA* amplification [29].

### 2.6. PTEN—The Phosphatase and Tensin Homolog Deleted on Chromosome Ten

As mentioned above, *PTEN* mutations are characteristic for endometrioid carcinomas [75]. *PTEN* is a tumor suppressor gene located on chromosome 10q23.3. Loss of function, often due to somatic or germline mutations, has been found in various tumor types and is responsible for the development of endometriosis [76]. *PTEN* is an inhibitor of PI3K/AKT signaling that controls the rate of cell division and promotes apoptosis [77]. Germline mutations in *PTEN* lead to Cowden syndrome. In ovarian cancer, gene deletion is a common *PTEN* mutation generating a protein-deficient state with a complete loss of functional phenotype. Nevertheless, the *PTEN* mutation’s definitive role in ovarian cancer susceptibility is still under debate [78,79,80,81]. Epithelial ovarian cancer type I, characterized by *KRAS*, *BRAF*, *PTEN*, and beta-catenin mutations, typically arises from precursor lesions like endometriosis. Somatic *PTEN* mutations, combined with *KRAS* mutations, may predispose to invasive and metastatic endometrioid ovarian cancer. Targeted therapies for these mutations in ovarian cancer type I have been unsatisfactory [82,83]. Ovarian cancer risk-reducing surgery is not recommended for *PTEN* pathogenic variant carriers, but they face an increased risk of endometrial cancer (approximately 5–10%) [76]. The inactivation of *PTEN* may also play a role in mediating resistance to PI3K inhibition as long as *PTEN* and *PIK3CA* alterations co-occur in some ovarian carcinoma patients [29].

### 2.7. CTNNB1 Mutation

*CTNNB1* (β-Catenin-1) mutations in ovarian cancer refer to genetic alterations in the *CTNNB1* gene that encodes the beta-catenin protein. Beta-catenin is a crucial component of cell adhesion structures and is also involved in the WT signaling pathway, which plays a role in regulating cell proliferation and differentiation [84]. Mutations in the *CTNNB1* gene can lead to abnormal activation of the above-mentioned signaling pathway, contributing to uncontrolled cell growth and the development of cancer. In ovarian cancer patients, *CTNNB1* mutations are often associated with specific subtypes, such as ovarian clear cell carcinoma and endometrioid carcinoma. The study conducted by Liu et al. identified mutations in *CTNNB1* exon 3 as a potential risk factor for recurrence in low-grade, early-stage endometrioid carcinoma and may be involved in the development of some mucinous-type ovarian carcinomas [85,86]. Subsequent research has confirmed this association in the majority of studies, although not universally across all investigations [85,87,88]. Lastly, a strong association of *CTNNB1* and *KRAS* mutations linking genotype to the type of recurrence was demonstrated in low-grade endometrioid endometrial carcinoma [89].

### 2.8. C-myc Mutation

C-myc stands out as one of the frequently amplified or overexpressed oncoproteins in ovarian cancer: it was identified in 30–60% of patients [90]. Its pivotal role in driving the initiation and progression of ovarian cancer underscores its significance [91]. Several studies suggest that c-myc amplification may serve as a prognostic indicator, signaling a more aggressive course of disease and potentially poorer outcomes in ovarian cancer patients [92]. Targeting c-myc directly is challenging due to its intricate role in cellular processes [93]. Nevertheless, researchers are exploring therapeutic strategies that could inhibit c-myc activity or downstream signaling pathways as a potential tool for treatment [94], especially to decrease the activity of ovarian cancer stem cells [95]. Ongoing research and clinical trials aim to expand our understanding of c-myc amplification in ovarian cancer. This involves an investigation of its molecular mechanisms and an exploration of targeted therapeutic approaches tailored to the specific context of c-myc alterations [96]. Specifically, c-myc’s influence extends to the polyamine pathway, with a profound impact on polyamine metabolism, a process intricately linked to ovarian malignancy. Special attention is given to the potential therapeutic approach offered by a direct focus on c-myc-driven polyamine metabolism for the treatment of ovarian cancers [97]. Additionally, the identification of polyamine signatures in biofluids is highlighted for potential applications in the early detection of ovarian cancer [97]. At present, various approaches exist for disrupting the activity of c-myc that include inhibiting cofactors recruited by c-myc or employing strategies that influence epigenetic mechanisms associated with c-myc [94,98,99].

With the knowledge that genetic background has a significant impact on the treatment protocols for women with ovarian cancer, new protocols are continuously being tested and integrated into treatment regimens. In the past twenty years, new technologies that selectively identify therapeutic targets have been developed, and the employment of immunotherapy could be a breakthrough in the treatment of various types of cancer, including ovarian carcinoma. Many clinical trials registered at https://clinicaltrials.gov have examined neoadjuvant or adjuvant chemo/immunotherapy regimens or immunotherapy alone in ovarian cancer patients in combination with radical ovariectomy. Table 1 presents examples of currently conducted/ongoing clinical studies on genomic mutations and therapeutic approaches in patients with ovarian cancer.

## 3. Genetic Considerations in Ovarian Cancer: From Hereditary Syndromes to Surgical Strategies

Overall, understanding inherent genetic functions is important for more precisely matching patients whose cancers harbor those mutations with cognate drugs to optimize response and outcome. As an increasing body of evidence emphasizes distinct developmental origins and molecular pathogenesis among OC subtypes, there is a growing acknowledgment that the above-mentioned five histologically defined groups constitute separate disease entities. Consequently, there is a recognized imperative for stratification in both clinical and research settings [100].

Hereditary cancer syndromes are associated with genetic disorders, and *BRCA1/2* mutations are among the most common. There is an increased risk observed of breast cancer in women and men, and ovarian cancer, fallopian tube cancer, primary peritoneal cancer, and malignant prostate and pancreatic cancer. The possibility of using non-invasive methods effectively in OC screening has not been proven [101]. The presence of genetic abnormalities within *BRCA1* increases the risk by up to 44% and in the case of *BRCA2* by up to 17% in patients at the age of 80 or younger [102]. Due to the inability to effectively screen for ovarian cancer, global organizations including the American College of Obstetricians and Gynecologists recommend the simultaneous preventive removal of both fallopian tubes and ovaries at the age of 35–40 in the case of *BRCA1* mutations and at the age of 40–45 in the case of *BRCA2* mutations [103]. Recommendations and guidelines regarding *BRCA1* and *BRCA2* mutations formulated by selected gynecological societies are included in Table 2. A systematic analysis of Cochrane Library 2018 data, as part of which 8087 cases of women with *BRCA1/2* mutations were assessed, showed lower mortality due to HGSC (HR 0.06, 95% CI 0.02 to 0.17; I^2^ = 69%; *p* < 0.0001) and longer overall survival (HR 0.32, 95% CI 0.19 to 0.54; *p* < 0.001) in patients undergoing risk-reducing salpingo-oophorectomy (RRSO) [104]. It should be emphasized that the authors suggested a high risk of bias and low quality of evidence, which requires further well-designed clinical trials. The scope of the surgical procedure depends on several variables. During its planning, the type of mutation, the patient’s age, the possibility of applying hormonal replacement therapy and maternity plans are important. RRSO is a method that reduces the risk of OC, but some candidates for that procedure do not consent to the simultaneous removal of the ovaries and fallopian tubes due to the side effects of iatrogenic menopause. It is possible to experience vasomotor disorders and sexual and cognitive dysfunction, and there is an increased risk of osteoporosis. Therefore, it has been proposed to perform a two-stage operation—radical fimbriectomy (RF) and delayed oophorectomy (DO). The first stage is the removal of the fallopian tube, along with the part of the ovary adjacent to the tubo-ovarian junction. A bilateral oophorectomy is performed in patients at the age of 50 or older, or after menopause if it occurs earlier. A prospective study was conducted with the participation of 119 patients with confirmed *BRCA 1/2* mutations who refused RRSO due to potential side effects. Forty-six patients underwent DO; the median age was 46 years. No differences in mortality were found among patients treated using RRSO and RF/DO [105]. Factors that may limit the application of RF/DO are oncological safety and the risk of potential side effects of the surgery. In 2023, a meta-analysis summarizing the potential variables considered when deciding whether to perform one- or two-stage surgery was published [106]. The decisions are made on a case-by-case basis; strict guidelines are not known. Based on the systematic review presented in 2020, OC occurred in 1.2% of cases during RRSO [107]. The mean age of patients was 53.2 years (42.4 to 67 years old). A systematic review of RRSO performed among the Chinese population showed an incidence of serous tubal intraepithelial carcinoma (STIC) and OC of 1% and 3%, respectively, which is 200 times higher than the population risk [108]. The advantage of applying RRSO, in addition to reducing the risk of possible OC, is the chance to diagnose STIC and OC which, due to the lower degree of clinical advancement, increases the patient’s chance of recovery. Genetic abnormalities within *BRCA* 1 and *2* increase the risk of ovarian and fallopian tube cancer, but also of primary peritoneal cancer (PC). Further research is necessary to clarify the pathogenesis of PC. The presence of STIC found during RRSO increases the risk of PC in the future. The proposed pathogenetic mechanism involves the formation of HGSC on the background of STIC. To determine the risk of PC in a patient diagnosed with STIC during RRSO, a meta-analysis and systematic review were conducted based on 17 studies and a total of 3121 patients diagnosed with STIC during RRSO. The risk was 33.9% (*p* < 0.001) compared to patients who were not diagnosed with STIC during prophylactic surgery. The 5-year risk was 10.5% and the 10-year risk was 27.5%, respectively, compared to 0.9% for patients without STIC [109]. Surgical procedures should be tailored individually depending on the type of genetic mutation, the patient’s age, the possibility of hormone replacement therapy, maternity plans, and the patient’s individual surgical risk.

## 4. Conclusions

In conclusion, the recognition of distinct developmental origins and molecular pathogenesis among ovarian cancer subtypes underscores the imperative for genetic stratification. Understanding the inherent genetic function is crucial for effectively pairing patients with specific mutations to appropriate drugs, optimizing the treatment response and outcomes. The identification of separate disease entities within histologically defined groups further emphasizes the need for tailored approaches in both clinical and research settings, contributing to the ongoing evolution of precision medicine in ovarian cancer.

## Figures and Tables

**Table 1:** Gene mutations and indications for the chemo-/immunotherapy of ovarian carcinoma patients.

Mechanism of Action	Phase	Therapeutic Intervention	NCT Number	Gene Mutation
Adavosertib is an inhibitor of Wee1-like protein kinase; Paclitaxel targets microtubules; Carboplatin inhibits DNA synthesis.	Phase 2	Adavosertib (MK-1775) Paclitaxel Carboplatin	NCT01357161	*TP53*
Ganetespib is a second-generation Hsp90 inhibitor; Paclitaxel targets microtubules.	Phase 1 Phase 2	Ganetespib Paclitaxel	NCT02012192	
Lunresertib is an inhibitor of PKMYT1; Carboplatin inhibits DNA synthesis; Paclitaxel targets microtubules.	Phase 1	Lunresertib (RP-6306) Carboplatin Paclitaxel	NCT06107868	
SRA737 is an inhibitor of Chk1; Gemcitabine inhibits DNA synthesis; Cisplatin inhibits replication and transcription of DNA.	Phase 1 Phase 2	SRA737 Gemcitabine Cisplatin	NCT02797977	
PARP inhibitors	Phase 2	Olaparib	NCT01078662	*BRCA1*/ *BRCA2*
Small molecule selectively killing HR-deficient cancer cells through the binding and stabilization of the G4 DNA structure.	Phase 1	CX-5461	NCT04890613	
Durvalumab is blocking the action of PD-L1; Olaparib is a PARP inhibitor; Tremelimumab turns off the inhibitory mechanism and allows CTLs to continue to destroy the cancer cells.	Phase 2	Durvalumab Olaparib Tremelimumab	NCT02953457	
PARP inhibitors	Phase 2	Veliparib (ABT-888)	NCT01540565	
Copanlisib acts as an inhibitor of PI3K; Niraparib acts as a PARP inhibitor.	Phase 1	Copanlisib Niraparib	NCT03586661	
ERK1/2 inhibitor.	-	Ulixertinib (BVD-523)	NCT04566393	*KRAS* and *BRAF*
Avutometinib inhibits both the phosphorylation of MEK by RAF and the activation of ERK1/2 by MEK; Defactinib acts as an inhibitor of PTK2 kinase.	Phase 2	Avutometinib (VS-6766) Defactinib	NCT04625270	
Bevacizumab acts by selectively binding circulating VEGF; Niraparib acts as PARP inhibitor.	Phase 2	Bevacizumab Niraparib	NCT05523440	*ARID1A*
PLX2853 acts as an inhibitor of BET domain proteins; Carboplatin inhibits DNA synthesis.	Phase 1 Phase 2	PLX2853 Carboplatin	NCT04493619	
NXP800 acts as an inhibitor of HSF1 pathway.	Phase 1	NXP800	NCT05226507	
Dasatinib inhibiting of Src tyrosine kinase.	Phase 2	Dasatinib	NCT02059265	
Copanlisib acts as an inhibitor of PI3K; Fulvestrant acts as a selective estrogen receptor degrader.	Phase 2	Copanlisib Fulvestrant	NCT05082025	*PIK3CA*
Risovalisib acts as an inhibitor of PI3K.	Phase 1	Risovalisib (CYH33)	NCT04586335	
Miransertib acts as a selective Akt inhibitor; Carboplatin inhibits DNA synthesis; Paclitaxel targets microtubules; Anastrozole is an antiestrogenic medication.	Phase 1	Miransertib (ARQ 092) Carboplatin Paclitaxel Anastrozole	NCT02476955	
STX-478 acts as an inhibitor of PI3K; Fulvestrant acts as a selective estrogen receptor degrader.	Phase 1 Phase 2	STX-478 Fulvestrant	NCT05768139	
Copanlisib acts as an inhibitor of PI3K; Fulvestrant acts as a selective estrogen receptor degrader.	Phase 2	Copanlisib Fulvestrant	NCT05082025	*PTEN*

Abbreviation: PKMYT1—membrane associated tyrosine/threonine 1 protein kinase; Chk1—checkpoint kinase 1; PARP—poly ADP ribose polymerase; PD-L1—programmed death receptor ligand—1; CTLs—cytotoxic T lymphocytes; PIK3—phosphatidylinositol-3-kinase; ERK1/2—extracellular signal-regulated kinase ½; MEK—mitogen-activated protein kinase; RAF—rapidly accelerated fibrosarcoma; PTK2—protein tyrosine kinase 2; VEGF—vascular endothelial growth factor; BET—bromodomain and extra-terminal; HSF—heat shock factor 1.

**Table 2:** Guidelines for surgical management in BRCA mutation carriers.

BRCA 2	BRCA 1	The Name of the Treatment Organization
Reasonable to delay RRSO until age 40–45	RRSO at age 35–40 or after reproduction	NCCN (National Comprehensive Cancer Network)
RRSO after childbearing is completed or at age 40–45	RRSO after childbearing is completed or at age 35–40	ESMO (European Society of Medical Oncology)
RRSO at age 40–45	RRSO after childbearing is completed or at age 35–40	American College of Obstetricians and Gynecologists
5 years before the earliest recorded age of onset of ovarian cancer in the family or at age 40–45	5 years before the earliest recorded age of onset of ovarian cancer in the family or at age 35–40	Royal College of Obstetricians and Gynecologists
RRSO at age 40–45	RRSO at age 35–40	Society of Obstetricians and Gynaecologists of Canada

Abbreviation: RRSO—risk-reducing salpingo-oophorectomy.
